# Mechanisms controlling bacterial infection in myeloid cells under hypoxic conditions

**DOI:** 10.1007/s00018-020-03684-8

**Published:** 2020-10-30

**Authors:** Inaya Hayek, Valentin Schatz, Christian Bogdan, Jonathan Jantsch, Anja Lührmann

**Affiliations:** 1 Mikrobiologisches Institut, Klinische Mikrobiologie, Immunologie und Hygiene, Universitätsklinikum Erlangen, Friedrich-Alexander-Universität (FAU) Erlangen-Nürnberg, 91054 Erlangen, Germany; 2 Institut für Klinische Mikrobiologie und Hygiene, Universitätsklinikum Regensburg, Universität Regensburg, 93053 Regensburg, Germany; 3 Medical Immunology Campus Erlangen, FAU Erlangen-Nürnberg, 91054 Erlangen, Germany

**Keywords:** Hypoxia, HIF1α, Infection, Bacteria, Metabolism, Macrophages, Neutrophils, Dendritic cells

## Abstract

Various factors of the tissue microenvironment such as the oxygen concentration influence the host–pathogen interaction. During the past decade, hypoxia-driven signaling via hypoxia-inducible factors (HIF) has emerged as an important factor that affects both the pathogen and the host. In this chapter, we will review the current knowledge of this complex interplay, with a particular emphasis given to the impact of hypoxia and HIF on the inflammatory and antimicrobial activity of myeloid cells, the bacterial responses to hypoxia and the containment of bacterial infections under oxygen-limited conditions. We will also summarize how low oxygen concentrations influence the metabolism of neutrophils, macrophages and dendritic cells. Finally, we will discuss the consequences of hypoxia and HIFα activation for the invading pathogen, with a focus on *Pseudomonas aeruginosa*, *Mycobacterium tuberculosis*, *Coxiella burnetii*, *Salmonella enterica* and *Staphylococcus aureus*. This includes a description of the mechanisms and microbial factors, which the pathogens use to sense and react to hypoxic conditions.

## Introduction

Myeloid cells are the first line of defense against bacterial infections. They are equipped with an arsenal of mechanisms to prevent spreading of the intruders, to alert the adaptive immune system, to prevent bacterial proliferation and to eliminate the pathogens without inducing immunopathology (reviewed: [1]). In recent years, it has become clear that the interplay between myeloid cells and the pathogen is strongly affected by the (patho)physiological conditions prevailing at the site of infection. Thus, tonicity, availability of nutrients and oxygen tension significantly influence the outcome of the host–pathogen interaction [2–5]. The oxygen availability is of particular interest in this context. Several important antimicrobial effector pathways require oxygen, such as the phagocyte NADPH-oxidase (PHOX), which generates reactive oxygen species (ROS), and the inducible or type 2 nitric oxide synthase (iNOS or NOS2), which produces high amounts of nitric oxide (NO) and leads to the formation of subsequent reactive nitrogen species (RNS). Both ROS and NO are capable of damaging and killing bacterial microorganisms and, therefore, are important to control the infection [6]. In addition, it is well established that oxygen levels differ in various organs (reviewed: [7]). Even under resting conditions, the oxygen level of the renal medulla, skin, and bone marrow are low [8–10]. In these organs, the availability of oxygen and, hence, the tissue oxygenation is thought to be largely dependent on the organ-specific vascular network. Not only vascularization and supply of oxygen, but also the consumption rate influences the oxygen level available within the tissue. Infiltration of immune cells in an organ increases the consumption of oxygen and as a consequence reduces the available oxygen level [11]. Similarly, the low oxygen levels found in epithelial layers facing the gastrointestinal lumen [12, 13] result from the metabolism of gastrointestinal microbiota [14] and the action of their products on the host epithelium [15]. These latter examples demonstrate that already under resting conditions, bacteria have an impact on the oxygen availability in host tissue. Importantly, infections with microbial pathogens lead to oxygen consumption in the affected tissues, which influences the host as well as the pathogen and their interplay and, thus, the outcome of infection.

In the following, we will review (i) the basic methodology to measure oxygen in tissues; (ii) the principle impact of infections on tissue oxygen levels; (iii) the metabolism of innate immune cells under hypoxic conditions; (iv) the role of hypoxia-inducible factors (HIF); (v) the functional regulation of myeloid cells by hypoxia and HIF; (vi) the sensing of hypoxia by bacteria and their reaction to oxygen deprivation; and (vii) the mechanisms of control of bacterial infections under hypoxic conditions.

## Methods to quantify tissue oxygenation

Progress in our understanding of tissue oxygenation is limited by the fact that quantification of tissue oxygen is a difficult and tedious task (reviewed: [16, 17]). Over the last decades, different studies either employed the Clark polarographic electrode technique, used histochemical staining techniques to detect severely hypoxic regions with the help of 2-nitroimidazole derivatives (e. g., EF5, piminidazole, CCI-103F) or applied luminescence-based technologies to monitor tissue oxygen levels (reviewed: [16]). More recently, positron emission tomography (PET), single-photon emission computed tomography (SPECT) and magnetic resonance imaging (MRI)-based technologies have become available and offer new opportunities to assess oxygen levels in inflamed and infected tissues [18–21]. The advantage of these methods is that they are noninvasive and do not cause tissue injuries. PET/SPECT/MRI entered preclinical and clinical application in late 2000s/early 2010s for oxygen quantification. These methods rely on administration of various hypoxia tracers that enable in vivo oxygen measurement without tissue destruction (reviewed: [21]). Several studies were performed using [^18^F]fluoromisonidazole ([^18^F]FMISO), which is often referred to as a “gold standard” in PET/SPECT. However, several disadvantages of [^18^F]FMISO led to the development of novel tracers, such as [^64^Cu][Cu-diacetyl-bis(N(4)-methylthiosemicarbazone)] ([^64^Cu][Cu(ATSM)]), ^68^ Ga-labeled tracers, technetium-99 m ([^99^mTc]Tc-BRU59-21, [^99^mTc]Tc-EDTA-2-MN) or molybdenum-99 co-labeled nitroimidazole-containing or nitroimidazole-free compounds [22–27].

## Infection triggers low tissue oxygen levels: underlying mechanisms

There is substantial evidence that inflamed and infected tissue displays low oxygen levels (reviewed: [16]). However, only recently, studies on the mechanisms that account for the reduced oxygen levels in infected and inflamed tissues were conducted. In a mouse model of pyelonephritis, inflammation-induced clotting contributed to a low oxygen microenvironment in the kidney [28]. Moreover, inflammation was able to trigger clotting processes and vice versa (reviewed: [29]). Clotting of vessels in infected tissues can help to sequester and compartmentalize infections [30], but will result in reduced oxygen levels in the afflicted tissues. Thus, induction of low tissue oxygenation might be a side effect of host efforts to inhibit bacterial spreading.

The influx of neutrophils also plays a role in mediating low tissue oxygenation upon an infection. In a seminal study, Campbell et al. demonstrated in a model of dextran sulfate sodium (DSS)-induced colitis that the influx of polymorphonuclear neutrophils (PMN) and their NADPH oxidase activity caused increased oxygen consumption and ultimately low mucosal tissue oxygenation [11]. As Campbell et al. used DSS to induce colitis and not a specific pathogen, it is formally unclear whether infections with intestinal pathogens induce low oxygen levels in mouse mucosal gut tissue. However, infections with enteropathogenic bacteria such as *Salmonella* and *Shigella* were shown to trigger low oxygen levels in infected lamina propria and serosal gut tissue [31–34]. The exact mechanisms that lead to low oxygen levels in *Salmonella*-infected tissues are still unknown. It is possible that *Salmonella* inhibits inflammation-triggered de novo formation of blood vessels by limiting vascular endothelial growth factor (VEGF)-driven angiogenesis [35]. In a *Shigella* infection model, the relative contribution of the oxygen consumption induced by enteropathogens and/or the infection-triggered inflammation was investigated. Tinevez and coworkers found that, unlike the above-mentioned *Salmonella* studies, the pathogen themselves, but not the infiltrating PMN, were largely responsible for the low oxygen conditions in the gut mucosa [34].

Nonetheless, in addition to the above-mentioned study, analyses in other models confirmed that PMN critically contributes to low tissue oxygen levels. The influx of PMN reduced local oxygen levels in a preclinical model of Herpes simplex virus 1 (HSV-1) keratitis [36]. Similarly, accumulation of PMN into a *Candida albicans-*induced subdermal abscess triggered a hypoxic microenvironment [37]. In addition, interleukin 1 (IL-1)-dependent signaling elicited low O_2_ levels in a preclinical model of pulmonary aspergillosis [38]. These findings suggest that the inflammatory response of the host and especially the activities of neutrophils are the main drivers of infection-triggered low tissue oxygenation .

Thus, from a mechanistic point of view, the reduced oxygen levels in infected tissues can result from several, presumably overlapping processes: (i) oxygen consumption by the invading immune cells; (ii) oxygen consumption by the invading pathogens; (iii) induction of host cell signaling cascades that control oxygen availability, by bacterial compounds and/or by host cell products; (iv) alterations of the microenvironment during the infection that result in changes in metabolism and thereby oxygen availability.

## Metabolic response of innate immune cells to hypoxia

**Fig. 1:**
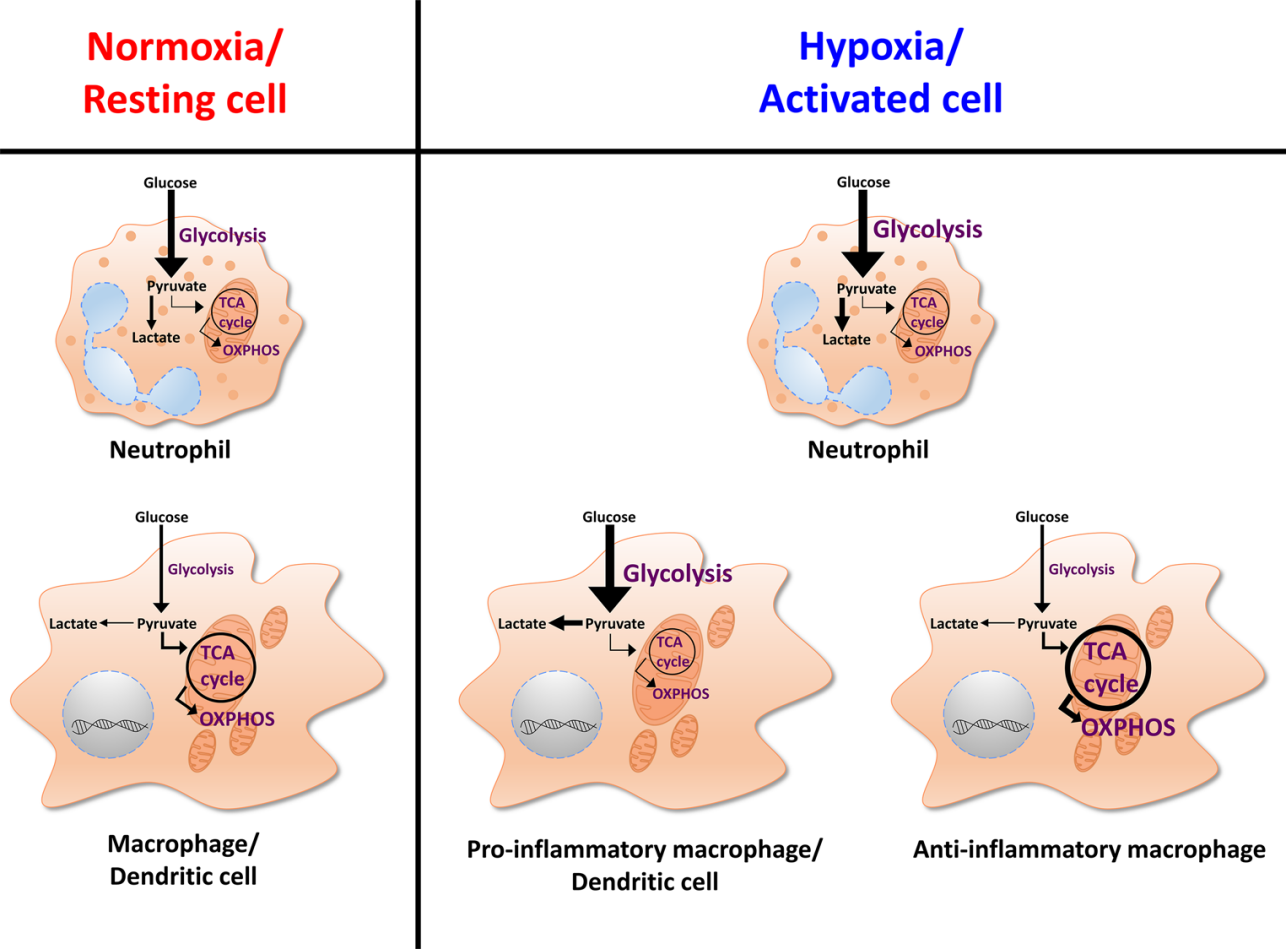
Cellular metabolism differences under normoxia and hypoxia or between resting or activated profiles of immune cells. Regardless of the oxygen or activation status, neutrophils witness increased glycolysis and decreased tricarboxylic acid (TCA) cycle and oxidative phosphorylation (OXPHOS) activity, with an augmentation of glycolysis under hypoxia or upon activation. Resting or normoxic macrophages/dendritic cells, however, depend on the TCA cycle to ensure longevity and biomass. Upon exposure to hypoxia or pro-inflammatory signals, they switch to heavy glycolysis and hamper the TCA cycle activity as well as OXPHOS. Macrophages, which have encountered anti-inflammatory signals, on the other hand, depend greatly on the TCA cycle activity and OXPHOS

Similar to other immune cells, myeloid cells require various cellular metabolic pathways to function and respond properly (reviewed: [39], Fig. 1). The prevailing metabolic pathways vary between different types and activation statuses of cells. Moreover, depending on the local microenvironment in which the cells reside, they must adapt their cellular metabolism (reviewed: [40]).

As discussed above, oxygen is limited in inflamed and infected tissues. Therefore, immune cells have to operate under hypoxic conditions and, consequently, switch to anaerobic glycolysis (reviewed: [41]), while the oxygen-dependent energy generation via the tricarboxylic acid (TCA) cycle is suppressed (reviewed: [39, 42–47]).

However, not only hypoxic immune cells rely on glycolysis for energy generation, but in general, activated or proliferating immune cells also shift their metabolism to aerobic glycolysis, which resembles a metabolic profile observed by Otto Warburg in tumor cells (Warburg effect) (reviewed: [41, 42]). Instead of using oxidative phosphorylation (OXPHOS) for highly efficient adenosine triphosphate (ATP) production, proliferating cells tend to increase their glycolysis rate, which, however, only generates two ATP molecules per round of glycolysis (reviewed: [48]). To compensate for the reduced or absent mitochondrial OXPHOS, proliferating cells need to increase their glucose uptake and boost their glycolytic activity to meet the energy demand of proliferating cells (reviewed: [41, 48]). Since activation of immune cells under normoxic conditions already triggers a switch to glycolytic energy generation, these cells are well prepared to operate in inflamed areas, which are very likely to display low oxygen levels. The hypoxia-inducible factor (HIF) plays an important role not only for the adaptation of cells to oxygen-poor environments (reviewed: [49, 50]), but also under normoxic conditions (reviewed: [39, 42, 44–46, 51]). Therefore, we will very briefly review the molecular mechanisms that result in HIF stabilization in myeloid cells and we will describe the role of HIF in the immunobiology of neutrophils, macrophages, and dendritic cells.

## Hypoxia and inflammation-/infection-triggered HIF stabilization

HIF, initially discovered as oxygen-dependent complex for erythropoietin induction in the liver and kidney, is part of the PER-ARNT-SIM (PAS) protein subfamily of the basic helix-loop-helix (bHLH) family (reviewed: [52]). This dimeric transcription factor consists of two subunits (HIFα and HIFβ), which have to dimerize to attach to the promoter region of target genes harboring the hypoxia response element (HRE). There is only one HIFα isoform, but there are three closely related HIFß isoforms, HIF1α, HIF2α, and HIF3α (reviewed: [53]). HIF1α is considered a ubiquitous transcription factor. According to the Immgen Database (www.immgen.org), all myeloid cells are able to express HIF1α mRNA at medium to high range level [54]. In contrast, the expression of HIF2α is much more restricted. Thus, HIF2α is expressed in endothelial cells [55, 56], but is also present in some immune cells [57, 58]. It is known that various cells express HIF3α [59], but there is only very limited data on its expression in innate immune cells. While thioglycolate-elicited peritoneal macrophages express prominent amounts of HIF2α mRNA, the mRNA expression of both HIF2α and HIF3α in other myeloid cells is low [54].

### Hypoxic HIF stabilization

HIF activity is regulated by modulating the stability of its α-subunit. Under conditions of ample oxygen, HIF1α is readily hydroxylated at proline residues located in the oxygen-dependent degradation domain, which is present in all three HIFα isoforms. Hydroxylation of the HIFα isoforms are governed by a class of enzymes called prolyl hydroxylases (PHDs), which serve as cellular oxygen sensors. PHD-mediated HIF1α-hydroxylation is highly specific [60] and leads to ubiquitination of HIF1α by von Hippel–Lindau (VHL) E3 ubiquitin ligase, which ultimately targets HIF1α for proteasomal degradation. Of note, PHD requires the presence of its cofactor Fe^2+^ and the co-substrate 2-oxoglutarate for HIF1α hydroxylation (reviewed: [61]). In the absence of oxygen, its co-substrate or co-factors, the PHD activity is suppressed, which in turn triggers subsequent HIF1α activation (reviewed: [49, 50, 52, 61]).

HIFα governs the expression of a plethora of different genes involved in metabolism, immune system regulation, and in general cellular functions in response to hypoxia (reviewed: [39, 42, 44–46, 51]). A detailed global assessment of HIF1α and HIF2α binding sites in MCF7 breast cancer cells, for instance, revealed that many of these sites bound HIF1α and HIF2α equally well, while there were only very few sites that bound HIF2α exclusively [62]. It will require further endeavors to understand differential regulation of target genes by HIFα isoforms in various cell types [63], including immune cells.

### Inflammatory and infectious HIFα stabilization

In addition to hypoxia, other non-hypoxic stimuli are able to induce HIFα accumulation. This holds especially true for immune cells in which several inflammatory stimuli and cytokines are known to trigger HIFα accumulation (reviewed: [2, 7, 16, 64–66]. In line with this, there is evidence that a large array of human pathogens or microbial products are able to induce HIF1α accumulation even in the presence of O_2_ [67]. The most prominent and best studied pathogen-associated molecular pattern in this respect is lipopolysaccharide (LPS), a component of the cell wall of Gram-negative bacteria [68]. Under normoxic conditions, the LPS-triggered HIFα accumulation depends on nuclear factor (NF)-κB- and p42/44 MAPK-dependent signal transduction [69, 70]. Ultimately, this results in PHD inhibition via (i) enhanced ROS and RNS production [71–74], (ii) metabolic inhibition of PHD by succinate accumulation [75], and/ or (iii) decrease of availability of the PHD cofactor Fe^2+^ [3]. Adding another layer of complexity, HIF activation induced by LPS leads to a distinct and different response of myeloid cells compared to hypoxia-driven HIF activation [76]. The mechanisms that underlie this divergent response are still unclear and warrant further investigation (reviewed: [16]).

Recently, Solis et al*.* discovered a novel mechanism leading to HIF1α stabilization in innate immunity, which turned out to be very different from hypoxic or inflammatory HIF1α stabilization [77]. The authors studied how mechanosensation activates innate immunity. Immune cells-infiltrating sites of infection in the lung faced cyclical hydrostatic pressure [77, 78]. Monocytes detected this mechanical force via PIEZO1, a mechanically activated ion channel. Ca^2+^-influx by PIEZO1 subsequently triggered activating protein-1 (AP-1), which led to the transcription of endothelin-1 (*Edn1*). EDN1, in turn, caused HIF1α stabilization that upregulated pro-inflammatory genes and thereby facilitated monocyte-driven pathogen clearance [77].

In addition, bacterial virulence factors such as the *Bartonella henselae* adhesion A (BadA) and bacterial siderophores (Fe-chelating agents) are able to trigger normoxic HIF1α stabilization ([79–81]; Table 1).

**Table 1:** Examples of bacterial genes involved in response to hypoxia

Bacteria	Gene	Research model	Biological consequence	References
*Pseudomonas aeruginosa*	AQ signaling molecules	Infection of cell lines	Downregulated HIF1 protein levels	[82]
			Via 26S proteasomal degradation	
			VEGF secretion	
*Pseudomonas aeruginosa*	AtvR	Knock-out mutants;	Response to hypoxia; involved	[123]
		Cultivation in broth; infection of	in virulence	
		cell lines; in vivo infection in mice		
*Pseudomonas aeruginosa*	AdhA	Cultivation in broth	Increased expression under hypoxia	[143]
			Allowed growth on ethanol	
			Increased acetate production	
			Decreased of pH	
*Pseudomonas aeruginosa*	PPHD	Knock-out mutants;	Suppressed antibiotic resistance	[122]
		Infection of *Galleria mellonella*	and pathogenicity	
*Bartonella henselae*	BadA	Infection of cell lines	HIF1 activation	[80]
			Secretion of proangiogenic cytokines	
*Bartonella henselae*	Pili	Infection of cell lines	HIF1 activation	[81]
			VEGF secretion	
*Chlamyida pneumoniae*	CPAF	Cell-free degradation assays	Degraded HIF1	[83]
*Mycobacterium tuberculosis*	DosR regulon	Cultivation in broth	Allowed anaerobic survival	[148]
*Mycobacterium tuberculosis*	Rv0081	ChIP-Seq	Mediated response to hypoxia	[134]
*Mycobacterium tuberculosis*	Clp gene regulator (Rv2745c)	Cultivation in broth	Implicated in response to hypoxia	[153]
*Mycobacterium tuberculosis*	TreS	Growth in broth	Implicated in hypoxia-induced	[154]
		Infection of primary cells	Metabolic reprograming of *M. tb*	
*Mycobacterium tuberculosis*	Rv0998	Cultivation in broth	Acetylated DosR; negative influence	[131]
		Infection of primary cells	Adaption to hypoxia	
			Contributed to pathogenesis	
*Mycobacterium tuberculosis*	MtrB	Cultivation in broth	Allowed survival under hypoxia	[130]
		Infection of primary cells	Required for establishing infection	
		Infection in mice	Regulated DosR regulon	
*Salmonella enterica*	Sal (Siderophore)	Infection of cell lines	HIF1 activation	[79]
			VEGF secretion	
*Yersina enterocolitica*	Ybt (Siderophore)	Infection of cell lines	HIF1 activation	[79]
*Staphylococcus aureus*	SrrAB two-component system	Growth in broth	Allowed resistance to hypoxia	[162]

An unexpected observation was that certain pathogens promoted the degradation of HIF1α rather than causing HIF1α stabilization (Table 1). For instance, *Salmonella* interfered with HIF1α accumulation [35]. Moreover, *Pseudomonas aeruginosa* 2-alkyl-4-quinolone (AQ) quorum sensing signaling molecule directly targeted HIF1α for proteasomal degradation independently of PHDs [82]. Similarly, HIF1α was degraded during the late phases of intracellular chlamydial replication. In contrast, during the early phase of infection, *C. trachomatis* enhanced HIF1α stabilization [83].

Altogether, these findings already suggest that HIF1α is not only required for the cellular adaptation to hypoxia, but also for the immune response to infection under normoxic conditions. Therefore, for studying HIFα-responses in the context of infections, we need to take into account that the HIFα response may be triggered and manipulated by the pathogens itself (Table 1) and/or the low oxygen environment induced by the infection.

## Role of hypoxia and HIF in the immunobiology of neutrophils, macrophages, and dendritic cells

### Hypoxia and neutrophils

Both inflammatory and hypoxic HIF1α stabilization play a major role in activated neutrophils [84]. Since neutrophils require high ATP to combat infections, they depend on increased glycolysis to meet their energetic needs. Interestingly, neutrophils possess only a limited number of mitochondria, which, in addition, do not participate in the production of ATP, but are rather involved in regulating cell death decisions [42, 85]. Thus, neutrophils most likely depend on glycolysis for ATP-production. This assumption is supported by the observation that ATP-production was reduced in neutrophils treated with an inhibitor of glycolysis (2-deoxyglucose). In 2003, Cramer et al. showed that peritoneal neutrophils lacking HIF1α produced 40% less ATP than wild-type controls [86]. The fact that HIF1α, induced by hypoxia or by LPS stimulation, increases the expression of glycolytic target genes, including pyruvate kinase M2 (*Pkm2*), phosphoglycerate kinase (*Pgk*), glyceraldehyde 3-phosphate dehydrogenase (*Gapdh*), and triosephosphate isomerse-1 (*Tpi1*) [87–89], offers an explanation for the crucial role of HIF1α in ATP production.

Stabilization of HIFα in neutrophils using a PHD2-deficient mouse model revealed that HIFα is critically involved in augmented inflammatory and antimicrobial responses against *Streptococcus pneumoniae* through rapid recruitment, enhanced chemotaxis, and prolonged survival of neutrophils. Of note, stabilization of HIFα by interfering with PHD activity in neutrophils did not trigger changes in respiratory burst or in inner mitochondrial membrane potential [89]. However, hypoxic HIF1α enhanced production of granule proteases (neutrophil elastase and cathepsin G) and antimicrobial peptides (cathelicidin) in neutrophils [90]. In addition, HIF1α (but not hypoxia; see “Hypoxia-mediated containment of bacterial infections”) promoted the formation of neutrophil extracellular traps (NET) [91]. Normoxic [92] and hypoxic HIF1α also increased the lifespan of the otherwise short-lived neutrophils by inhibiting apoptosis via activating the NF-κB pathway [87]. This was accompanied by the hypoxia-induced release of the macrophage inflammatory protein-1β (MIP-1β) enhancing the survival effect of neutrophils under hypoxia [87].

Similar to HIF1α, HIF2α was able to prolong longevity of granulocytes as well, while having little impact on phagocytosis of bacteria [58]. Overall, these findings clearly demonstrate that HIFα is not only critical for maintaining the energy homeostasis of neutrophils, but, in addition, is critical for maintaining the longevity and inflammatory activity of neutrophils.

### Macrophages and hypoxia

Similar to neutrophils, macrophages also increase their glycolytic activity upon exposure to hypoxia and/ or inflammatory/ infectious conditions. In macrophages, HIF1α forms a complex with the pyruvate kinase M2 (PKM2) and thereby contributes to the upregulation of glycolytic activity of macrophages ( [75, 93], Fig. 1). Interestingly, HIF1α was not only involved in fostering metabolic reprogramming, but regulated the expression of pathogen-recognition molecules (e.g., toll-like receptor 4) [94], antimicrobial peptides [90], and of inducible NO synthase (NOS2) [90]. In addition, HIF1α activity was negatively correlated with IL-10 production [74, 95] and promoted the expression of IL-1β [75, 93], which ultimately helped fighting invading intruders [75, 86, 90, 93, 96–98].

In contrast to HIF1α, much less is known about the role of HIF2α in macrophages. Similar to HIF1α-knockout macrophages, HIF2α-deficient macrophages displayed reduced inflammatory responses compared to controls [57]. In contrast to HIF1α, however, HIF2α appears to be particularly important for the induction of regulatory and/or anti-inflammatory cascades. For instance, IL-4-activated macrophages expressed HIF2α, which induced arginase 1 and suppressed NO synthesis, while in classically activated macrophages, LPS-induced HIF1α raised the expression of NOS2 and thereby the generation of NO [99].

### Dendritic cells and hypoxia

As demonstrated earlier for neutrophils and macrophages, HIF1α stabilization contributed to increased glycolytic activities in activated mouse and human dendritic cells (DCs) [44, 100–103]. This supported the migration of DCs to lymph nodes to stimulate an immune response, which involves C–C chemokine receptor type 7 (CCR7) upregulation [104]. Inhibition of HIF1α [105] and HIF1α-dependent glycolytic activity impaired the migratory capacity of DC [106]. In addition, HIF1α was required for antigen presentation [107] and maturation [107, 108] and promoted DC-dependent T cell proliferation/activation [107–109]. Interestingly, an increase in glycolytic activity or stabilization of HIF1α alone was not sufficient to drive inflammatory outputs [108, 110]. However, under inflammatory conditions, HIF1α upregulated, in addition to glycolytic genes, a set of inflammatory genes, such as prostaglandin-endoperoxide synthase 2 (Ptgs2) and NOS2, which, in addition to HIF1α, co-depended on NF-κB [76]. Thus, the context of HIF1α activation is of critical relevance (reviewed: [16]). Recently, evidence was provided that the strength of the signal used to trigger inflammatory DC activation influences DC metabolism and their inflammatory output [104]. In contrast to weak DC activators such as “house dust mite-derived allergens”, a potent pro-inflammatory stimulus, such as LPS, led to a strong HIF1α-dependent pro-inflammatory phenotype, an increase in glycolysis and cessation of mitochondrial respiration. This is in line with earlier findings that the long-term commitment to glycolysis in activated DCs was indirectly regulated by PI(3)K-Akt-mTORC1 upregulation of NOS2 and HIF1α [111]. Moreover, conditions that resulted in excess glycolytic activity of DC even limited the ability of DC to induce T cell responses [73]. Weakly activated DCs, in contrast, showed no significant HIF1α accumulation, along with decreased pro-inflammation and absent glycolytic reprogramming [104]. Collectively, these findings suggest that fine-tuning of the immune-metabolism via HIF1α holds great potential in modifying DC immunobiology.

HIF1α is not only important for acute innate immune responses, but is also an important factor in trained immunity [112]. Trained immunity is a term for the memory-like response that is generated in innate immune cells due to a previous inflammatory stimulus which enables innate immune cells to respond more vigorously to a second insult in a nonspecific manner [113–115]. Trained immunity requires Akt-mTOR-HIF1α-dependent glycolytic reprogramming [114, 116]. 

However, boosting of HIF1α activity to fight infections bears risks as well. Recent evidence demonstrates that increasing HIF1α responses by subjecting mice to acute low oxygen environments aggravated inflammatory responses and can be detrimental. In contrast, long-term exposure to low oxygen environments dampened HIF1α activation, glycolysis and decreased overall pathology [117]. Further studies are required to understand the differences of HIF1α signaling in hypoxic preconditioning versus its role in trained immunity.

**Fig. 2:**
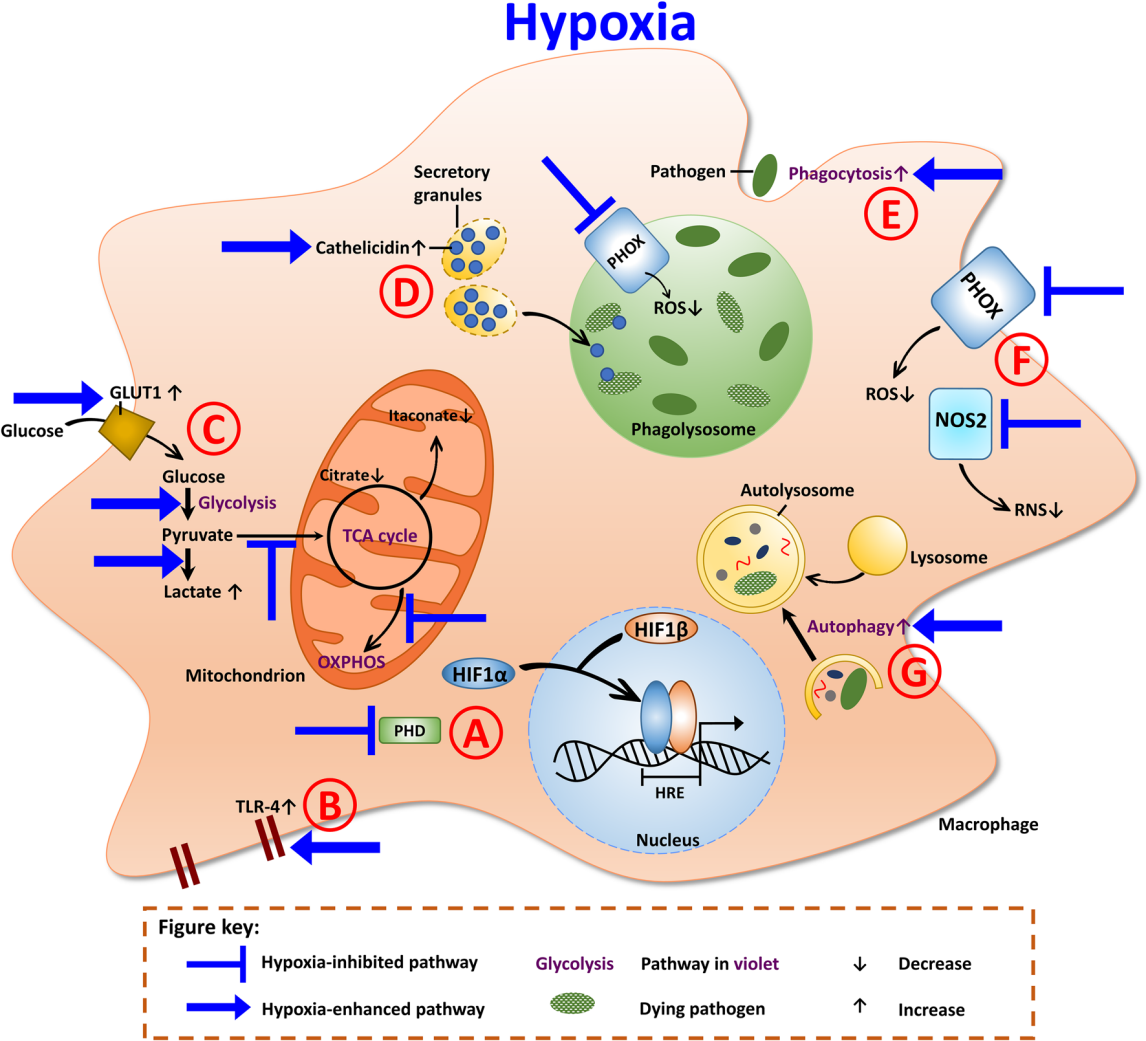
Antimicrobial mechanisms of macrophages under hypoxia. **a** Prolyl hydroxlases (PHD) are inactive in the absence of oxygen and thus, HIF1α is stabilized. HIF1α translocates into the nucleus, where it dimerizes with HIF1β. The dimer binds to the hypoxia responsive elements (HRE) and induces the transcription of target genes. **b** Hypoxia induces increased toll-like receptor 4 (TLR-4) expression. **c** Under hypoxia, the expression of glucose transporter GLUT1 is enhanced, which leads to increased glucose uptake into the cell. Hypoxia also enhances glycolysis. The end product of glycolysis, pyruvate, is metabolized into lactate. Due to the inhibition of the TCA cycle, less citrate and itaconate (the antimicrobial metabolite) are generated. Furthermore, hypoxia impairs OXPHOS. **d** Cathelicidin, the antimicrobial peptide, is also augmented under hypoxia. Cathelicidin can then be transported into the phagolysosome to eliminate pathogens. **e** The phagocytic uptake of macrophages is enhanced under hypoxia. **f** The oxygen-dependent effectors, PHOX and NOS2, are impaired under hypoxia. Thus, less ROS and RNS production is evident. **g** The process of autophagy is also increased under hypoxia

Collectively, neutrophils, macrophages and DCs are adept in accommodating to hypoxic niches by adjusting their inflammatory and metabolic activity (Fig. 2). HIF1α plays a key role in the regulation of this bidirectional interplay between metabolism and inflammation.

## Bacterial sensing of and reaction to hypoxia

### Bacterial oxygen sensing

Not only does the host sense hypoxia and react to it accordingly, but also the pathogen perceives and responds to drops in oxygen levels (Table 1). The bacterial response to hypoxia is species specific and depends on the genetic configuration. Enteric pathogens, for example, are capable of growing under low oxygen conditions (reviewed: [118]). However, even the obligative aerobic species of *Pseudomonas (P.) aeruginosa* is able to adapt to low oxygen levels and persist under these conditions [119]. To change transcription and translation, the pathogen has to be able to sense the altered environmental conditions. Thus, *P. aeruginosa* contains a homolog to the eukaryotic 2-oxoglutarate-dependent prolyl hydroxylases that are key oxygen sensors. This homolog was termed *Pseudomonas* prolyl-hydroxylase domain-containing protein (PPHD) and has been implicated in oxygen sensing [120, 121]. In addition, PPDH induces the expression of several virulence factors, and pharmacological repression of PPDH reduces the pathogenicity of *P. aeruginosa* in a mouse model of pneumonia [121]. The fact that PPDH also influences antibiotic susceptibility, expression of efflux pumps, motility and biofilm formation [122] demonstrates the importance of this oxygen sensor. *P. aeruginosa* encodes for additional oxygen sensors. One of them, the response regulator AtvR, seems to be important for survival under hypoxic conditions within the host [123]. Hypoxia influences *P. aeruginosa* transcription, which allows the pathogen to adapt metabolically [124].

Similarly to *P. aeruginosa*, *M. tuberculosis* grows better under normoxic conditions than in low oxygen environments [125]. Nonetheless, *M. tuberculosis* is capable of adapting to hypoxic environments. *M. tuberculosis* encodes for several two-component systems that allow the pathogen to respond to environmental cues, including oxygen. Two-component systems are composed of a sensor kinase and a cognate response regulator (reviewed: [126–128]). Recently, it was reported that an *M. tuberculosis* strain lacking the *M. tuberculosis* two-component regulatory system MtrA/MtrB (MtrAB) showed a decreased viability under hypoxia. MtrAB interacts with the non-cognate response regulator dormancy survival regulator (Dos)R and induces DosR-mediated gene expression. As DosR is part of the oxygen- and redox-sensing two-component system DosR/DosT [129], it allows adaptation to the hypoxic environment [130]. In detail, under hypoxia, DosR is acetylated at lysine 182 by the *M. tuberculosis* acetyltransferase Rv0998. This increases the DNA-binding ability of DosR to promote transcription of genes, which allows the adaptation to hypoxia [131]. Other bacterial transcription factors are also involved in hypoxic gene regulation [132, 133], from which Rv0081 seems to form the largest hub [134]. The DosR/DosT regulon not only helps the pathogen to adapt to the hypoxic environment, but it also modulates the host cell response to infection. The rv2626c gene, one of the most prominently induced genes of DosR/DosT regulon, is involved in inducing a pro-inflammatory host cell response and necrotic cell death [135]. As the DosR/DosT regulon is important for adaptation and survival of *M. tuberculosis* under hypoxic conditions, it might be a good target for new therapeutics. One promising inhibitor is artemisinin that disables the heme-base DosT sensor kinase [136]; further inhibitors with multiple distinct mechanisms have been identified [137].

### Bacterial responses to hypoxia: modulation of the host tissue response

Tobin and coworkers used a zebrafish model to investigate the role of tissue oxygen in the *Mycobacterium*–host interplay. They showed that mycobacterial granuloma in zebrafish also become hypoxic. In addition, they discovered that *M. marinum*-infected macrophages trigger a vascular endothelial angiogenic program in granulomas. This response was absent when a replication-deficient *M. marinum* strain was used that was lacking ESX1 protein export systems. These findings indicate that *Mycobacteria* manipulate angiogenesis of the host to generate an at least partially vascularized and hence oxygenated microenvironment that is required to allow mycobacterial replication [138]. Therefore, *M. marinum* is able to counteract the efforts of the immune system to withhold oxygen from the invading pathogen. Although *M. marinum* infection in zebrafish is only a surrogate model mimicking immune responses in tuberculosis, it has offered substantial insights into mycobacterial pathogenesis over the last decades [139]. The question how angiogenesis and tissue oxygenation are regulated is certainly also of central relevance for *M. tuberculosis* infections.

### Bacterial response to hypoxia: changes of the bacterial metabolism

Because the host metabolism changes with the level of oxygen, the pathogens also have to adapt to the altered metabolic environment (Table 1). This is especially important for pathogens that utilize host metabolites [140]. A recent report demonstrated that *P. aeruginosa* can grow on ethanol, produced by many other microbes, including *Klebsiella pneumoniae* [141, 142], as a sole carbon source in hypoxic settings. Accordingly, under hypoxic conditions, *P. aeruginosa* upregulates the NAD-linked alcohol dehydrogenase AdhA, which enables the pathogen to catabolize ethanol [143]. In addition, *P. aeruginosa* can respire nitrate and utilize pyruvate when oxygen is limited [144–146]. These changes in metabolism allow the pathogen to grow even under unfavorable conditions.

### Bacterial response to hypoxia: induction of bacterial dormancy

*Mycobacterium tuberculosis* is able to survive in a low oxygen environment by inducing a state of dormancy that prevents sterile immunity [147]. For the induction of the dormancy survival program, the DosR/DosT regulon is essential [148]. Importantly, persistent bacteria develop a thick outer layer that helps to restrict entry of the antibiotic rifampicin [149] and confers antibiotic resistance. This is in agreement with other findings showing reduced antibiotic sensitivity of hypoxia-induced persistent *M. tuberculosis* [150]. Additionally, hypoxia might induce an internal bacterial program that alters the composition and function of multidrug efflux pumps resulting in antibiotic resistance, as reported for *Pseudomonas aeruginosa* [151]. During *C. burnetii* infection, the lack of oxygen also seems to induce a state of dormancy [95], but this has to be studied in more detail. It is clear that hypoxia allows the initial containment of oxygen-dependent pathogens, but triggers the development of bacterial persistence and dormancy that impairs pathogen elimination and prepares the ground for chronic latent and eventually recrudescing infections.

Bacteria might re-encounter atmospheric oxygen levels and re-enter into replication mode. How this is controlled and regulated is not completely understood. In the case of *M. tuberculosis*, global transcriptional and physiological changes are required [152, 153]. The pathogen mounts a metabolic shift under hypoxic conditions, which allows accumulation of metabolites that can be used for growth after re-aeration [154].

## Hypoxia-mediated containment of bacterial infections

The antimicrobial and immunoregulatory enzyme NOS2, which produces high levels of RNS in macrophages [155], contributes to the control of *M. tuberculosis* [155, 156]. However, NOS2 requires oxygen as a substrate (reviewed: [16]) and loses its efficiency under hypoxic conditions (reviewed: [157]). Thus, hypoxia inhibits this important antimicrobial defense mechanism. In addition, low concentration of RNS might even support bacterial survival and adaptation. Nitrite was found to induce transcriptional alterations in *M. tuberculosis* that allowed the pathogen to withstand stress conditions [158].

**Fig. 3:**
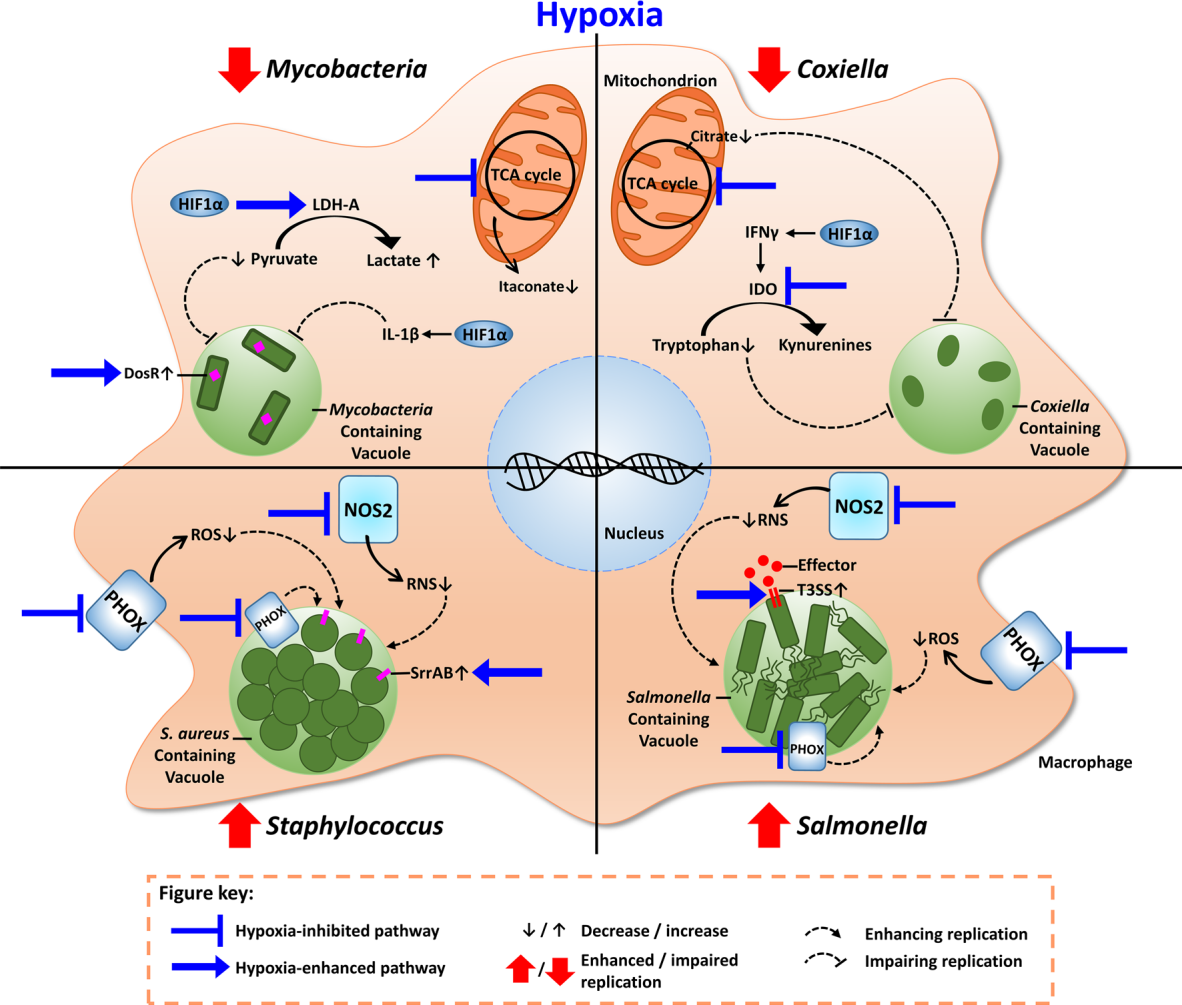
Host–pathogen interaction in macrophages under hypoxia. *Mycobacteria* replication is inhibited under hypoxia. This is due to HIF1α-mediated increase in lactate dehydrogenase (LDH-A), which catalyzes the conversion of the carbon source of *Mycobacteria*, pyruvate into lactate. Depletion of pyruvate results in reduced replication of *Mycobacteria*. HIF1α-induced IL-1β generation also limits *Mycobacteria* replication. Itaconate, an important antimicrobial effector, is decreased under hypoxia, due to hypoxia-mediated reduction of the TCA cycle. The DosR regulon is activated under hypoxia; this allows *Mycobacteria* to survive in those harsh conditions. *Coxiella* replication is also impaired under hypoxia. This is mainly due to reduced citrate levels. HIF1α-mediated increase in IFNγ results in augmentation of IDO, which catalyzes the transformation of tryptophan to kyurenines. This exhaustion of tryptophan limits its uptake by *Coxiella*, which is tryptophan auxotroph and, thus, replication is prevented. Yet, IDO is inhibited by hypoxia. *Salmonella* and *Staphylococcus*, however, are characterized by replicating under hypoxia. This is due to the inhibition of the antimicrobial effector enzymes, PHOX and NOS2, which leads to less ROS and RNS and, thus, aids replication. *Salmonella*’s virulence is enhanced under reduced oxygen levels, through increasing T3SS-dependent secretion of effector proteins; and Staphylococcus increases its two-component system SrrAB

Hypoxia does not only impair the antimicrobial activity of macrophages directed against *S. aureus*, but also blocks PHOX-dependent antimicrobial activity of granulocytes [159], such as NET formation [160] and degranulation of mast cells [161]. In the case of *S. aureus* infections, hypoxia is able to boost the virulence of *S. aureus* via the two-component system SrrAB ([162]; Fig. 3). In line with this, acute systemic hypoxia results in impaired antimicrobial response of infected mice kept under conditions of low oxygen [117, 163].

Therefore, the question arises how myeloid cells control bacterial infection under oxygen limiting conditions. For aerobic bacteria, the hypoxic microenvironment itself already impedes bacterial replication and, thereby, helps to control disease. In addition, the following mechanisms contribute to the control of bacterial infection under hypoxia: (i) induction of antimicrobial peptides; (ii) depletion of essential metabolites and (iii) alterations of the defense mechanisms for the benefit of the host (Table 1).

### Induction of antimicrobial peptides

Preclinical studies demonstrated that hypoxia was able to inhibit mycobacterial growth [164], as it caused the expression of antimicrobial molecules, like granulysin [165]. In line with this, the stabilization of HIF1α in bacterial dermatitis via lack of oxygen and/or infection itself resulted in the production and secretion of antibacterial peptides and pro-inflammatory cytokines (reviewed: [166]).

Hypoxia also helped to control infections with *Pseudomonas aeruginosa*. Increased bactericidal activities were observed in vitro and in vivo in a murine infection model [167]. HIF1α is partly responsible of this control, as demonstrated by the control of an ocular infection with *P. aeruginosa*, which might be dependent on NO and antimicrobial peptide production [168]. In a *Caenorhabditis elegans* infection model, loss of HIF1α enhanced the susceptibility of the nematode to *P. aeruginosa* [169]. Although HIF1α turned out to be important for the control of *P. aeruginosa* infections, the siderophore pyoverdin, an essential virulence factor of *P. aeruginosa* [170]*,* unexpectedly induced HIF1α stabilization [169]. However, under hypoxic conditions the expression of pyoverdin was limited [121]. Together, these data suggest that HIF1α stabilization is a relevant component of the host defense against *P. aeruginosa*  (Table 2).

**Table 2:** HIF1α and hypoxia-mediated effects in bacterial infections (examples)

Bacteria	Research model	HIF1α-mediated effect	Hypoxia-mediated effect	References
*Pseudomonas aeruginosa*	Growth in broth		Increased antibiotic resistance	[151]
			Expression of efflux pumps	
*Pseudomonas aeruginosa*	Infection of cell lines		Decreased internalization	[208]
*Pseudomonas aeruginosa*	Infection of mice		Contributed to disease control	[168]
			Regulated T cell infiltration	
			Enhanced cytokine and	
			antimicrobial peptide production	
*Pseudomonas aeruginosa*	Growth in broth		Suppression of siderophore and	[121]
			Exotoxin A production	
*Coxiella burnetii*	Infection of primary cells	Impaired STAT3 activation	Impeded replication	[95]
		Reduced citrate levels		
*Mycobacterium tuberculosis*	Infection of primary cells		Degreased intracellular growth	[164]
*Mycobacterium tuberculosis*	Infection of mice	Important for control of infection		[181]
		Regulated IFNγ-dependent immunity		
*Mycobacterium tuberculosis*	Infection of primary cells		Promotes granulysin expression	[165]
*Mycobacterium tuberculosis*	Growth in broth		Modulates bacterial	[154]
	infection of primary cells		metabolic pathways	
*Mycobacterium tuberculosis*	Growth in broth		Deacetylation of DosR; promotes	[131]
			adaption to hypoxia	
*Mycobacterium tuberculosis*	Infection of primary cells	Restricted growth via LDH		[171]
	Infection of mice	Expression and pyruvate reduction		
*Mycobacterium tuberculosis*	Infection of mice	Prevented leucocyte recruitment		[176]
*Mycobacterium marinum*	Infection of zebrafish	Decreased bacterial burden		[96]
		via iNOS induction		
*Mycobacterium marinum*	Infection of zebrafish		Promoted granuloma formation	[138]
			via VEGF induction	
*Mycobacterium marinum*	Infection of zebrafish	Decreased bacterial burden		[177]
		via IL-1β and NO production		
*Chlamydia pneumoniae*	Infection of cell lines		Enlargement of inclusions	[83]
	Degradation assay		Stabilized HIF1 in early infection	
			Degraded HIF1 in late infection	
*Chlamydia pneumoniae*	Infection of cell lines		Induced host cell glycolysis	[220]
			Allowed bacterial replication	
*Chlamydia trachomatis*	Infection of cell lines		Abrogated IFNγ-mediated	[187]
			anti-chlamydial activity	
*Shigella*	Growth in broth		Reduced effector secretion via T3SS	[13]
	Infection of cell lines		Enhanced invasion	
*Shigella*	in vivo and in vitro		Reduced effector secretion via T3SS	[34]
			Required for tissue colonization	
*Salmonella* Typhimurium	Infection of primary cells		Promoted replication	[33]
			Enhanced effector secretion via	
			SPI-2-T3SS	
*Yersinia enterocolitica*	Infection of mice	Reduced susceptibility		[79]
	Infection of cell lines	VEGF transcription		
Group A s*treptococci*	Infection of mice	Reduced skin lesion		[8, 90]
		Bacterial killing		

### Depletion of essential metabolites

Increased HIF1α levels correlated with bacterial killing in a zebrafish model of *M. avium* infections [96]. How the containment of mycobacteria is achieved via HIF1α accumulation is not yet clear. The underlying mechanism might involve HIF1α-mediated expression of the lactate dehydrogenase-A (LDH-A), which converts pyruvate to lactate. As *M. tuberculosis* can use pyruvate as a carbon source for intracellular replication, depletion of pyruvate might help to control mycobacteria ([171]; Fig. 3; Table 2).

The cytosolic conversion of citrate to acetyl-CoA is required for fatty acid biosynthesis and the synthesis of pro-inflammatory mediators [172–174]. Infection with *C. burnetii*, *L. pneumophila* [95] and *C. trachomatis* [175] induced an upregulation of citrate levels, which might result in a pro-inflammatory environment to fight the infection. However, *C. burnetii* only replicated in the presence of citrate ([95]; Fig. 3; Table 2), demonstrating that the increased availability of citrate in an inflammatory environment is exploited by the pathogen for its own purposes. Why *C. burnetii* requires citrate is unknown. It might need citrate for its energy metabolism. Alternatively, *C. burnetii* might sense host cell-derived citrate allowing the pathogen to adjust to the microenvironment and/or to express virulence genes. Importantly, citrate levels were markedly reduced under hypoxia, which resulted in impaired *C. burnetii* replication [95]. Thus, hypoxia-mediated restriction of citrate functions as a nutritional antibacterial effector mechanism.

### Modulation of host defense mechanisms: induction of pro-inflammatory cytokines

HIF1α stabilization drives expression of pro-inflammatory cytokines [75, 93]. In addition, HIF1α stabilization by oxygen deficiency or infection might enhance wound healing and tissue repair [166]. Experiments with mice deficient in HIF1α in the myeloid lineage support the assumption that HIF1α is required to prevent immuno-pathological consequences for the host. Thus, mice lacking HIF1α in myeloid cells showed a stronger inflammatory response and died earlier than wild-type mice during chronic *M. tuberculosis* infection [176]. HIF1α not only prevents immuno-pathology during infection, but also protects against *M. marinum* infection by inducing the pro-inflammatory cytokine IL-1β ( [177]; Fig. 3; Table 2).

Interferon  γ (IFNγ) is essential for the activation of macrophages and the control of many intracellular pathogens, including *M. tuberculosis* [178–180]. HIF1α regulates ~ 50% of all IFNγ-inducible genes during *M. tuberculosis* infection [181]. In uninfected dendritic cells hypoxia enhanced the IFNγ-induced mRNA expression of indoleamine 2,3-dioxygenase (IDO), which converts tryptophan into kynurenines [182]. IDO is known to suppress proliferation and survival of lymphocytes under normoxia [183]. Reports about the function of IDO during infections mainly concentrated on IDO-mediated depletion of tryptophan, which impaired the replication of tryptophan auxotrophic pathogens such as *Chlamydia* species, *C. burnetii* and *Toxoplasma gondii* ([184–186]; Fig. 3). However, unlike the findings with non-infected dendritic cells [182], IDO mRNA expression and activity were diminished in IFNγ-stimulated *C. trachomatis*-infected HEp2 cells under hypoxia due to an impaired IFNγ-STAT1 signaling [187]. Thus, the definitive role of IDO for the control of infections in a hypoxic microenvironment requires further studies.

### Modulation of host cell defense mechanisms: phagocytosis and phagosome maturation

Previous studies have found that hypoxia increased the phagocytic activity of macrophages in an HIF1α-dependent manner [188]. Exposure of mice to hypoxia improved the uptake of *E. coli* by peritoneal macrophages [188].

HIF1α influences autophagy [189, 190]. Autophagy is an important process to sequester damaged organelles, protein aggregates, or bacteria in a double-membrane-bound vesicle, the autophagosome. The fusion of the autophagosome with lysosomes results in the degradation of the sequestered material and replenishment of the host cell nutrient pool by the degraded products [191, 192]. Depending on the bacteria, the interaction with the autophagic pathway might be either detrimental or beneficial. The overall effect of HIF1α on autophagy still awaits clarification, as there are conflicting reports in the literature. We and others found that HIF1α can activate autophagy and, as a consequence, bacterial degradation [190, 193]. In contrast, during *Histoplasma capsulatum* infection, HIF1α decreased autolysosome maturation. However, this also led to containment of the pathogen, as *H. capsulatum* exploited the autophagic pathway for its own survival [194]. Further research is required to understand the different effect of HIF1α and/or hypoxia on autophagy in the different infection models.

In summary, an infected host cell uses several mechanisms to fight invading bacteria under oxygen-limiting conditions (Table 2). While several pathways/factors have been already identified, other still await identification. One of these factors might be itaconate, which is generated from cis-aconitate by the cis-aconitate-decarboxylase (encoded by the immune-responsive gene 1 [IRG1]) and possesses antimicrobial activity [195–197]. Our data indicate that infection with *Legionella pneumophila* or *C. burnetii* resulted in an increased level of itaconate, supporting previous reports [196]. Its antibacterial activity is at least partially mediated by inhibition of the glyoxylate shunt, which is necessary for bacteria to survive intracellularly [198]. Several pathogens encode genes required for itaconate degradation. These genes are important for the intracellular survival of the corresponding pathogens [199], indicating an important role of itaconate in the containment of infections. However, hypoxia diminished itaconate levels otherwise induced by infection with *L. pneumophila* or *C. burnetii*. Importanly, we observed bacterial replication only under normoxia and, thus, in the presence of itaconate [95]. This indicates that the itaconate levels induced by the infection might be insufficient to prevent *C. burnetii* and *L. pneumophila* replication. Our data suggest that the itaconate levels correlate with the oxygen concentration during infection. Thus, it is currently unlikely that itaconate contributes to the control of intracellular pathogens under hypoxic conditions.

## HIF stabilization as therapeutic strategy for the control of infections in hypoxic tissues

As explained above, HIF1α stabilization is an important regulator of innate immune responses. Therefore, its pharmacological stabilization is a possible treatment strategy to boost the host defense against bacterial infection in an oxygen-independent manner [200]. To stabilize HIF1α, inhibition of prolyl hydroxylases is widely used (reviewed: [201]). These prolyl hydroxylase inhibitors lead to HIF stabilization, mimicking hypoxic effects and have, therefore, been described as potential therapeutic agents [200, 202].

The PHD inhibitor mimosine triggered bactericidal activity in in vitro studies using human phagocytes infected with *S. aureus* [203]. Mimosine treatment increased HIF1α levels and ameliorated the clinical course of mice infected subcutaneously with *S. aureus* [203]. AKB-4924, a more potent pharmacological compound, enhanced cutaneous innate defenses against bacterial infections as well [204]. Its stabilizing effect on HIF1α was essential for the enhanced bactericidal activity of phagocytes, which might partially depend on AKB-4924-mediated upregulation of antimicrobial peptides and/or pro-inflammatory cytokines [204]. Inhibition of PHD, and, thus, stabilization of HIF1α by AKB-4924 not only boosted cutaneous defenses, but also improved the host innate immune response against urinary tract infections [205] and protected against colitis induced bacteremia [206].

Another prolyl hydroxylase inhibitor is dimethyloxalylglycine (DMOG). This substance is well tolerated and is able to ameliorate experimental colitis [207]. Its beneficial effects are, however, not limited to gastrointestinal inflammatory disorders. DMOG reduced the cytotoxic effects of *P. aeruginosa* infection on epithelial cells by decreasing *P. aeruginosa* internalization [208] as well. Moreover, mice pretreated with DMOG 48 h prior to *P. aeruginosa* infection showed reduced mortality rates [208]. These results suggest that DMOG might be a possible adjunctive therapeutic option to combat *P. aeruginosa* infections.

Desferrioxamine, an iron chelator, also leads to HIF1α stabilization by inhibiting PHD enzyme activity [209]. Addition of desferrioxamine to human-derived macrophages alters their cellular metabolism by increasing glycolysis in a HIF1α-dependent manner [210] in uninfected, LPS-stimulated, and *M. tuberculosis*-infected macrophages. In early stages of *M. tuberculosis* infection, desferrioxamine increased the immunological function of macrophages by boosting IL-1β through HIF1α [210]. Therefore, desferrioxamine might serve as a possible adjunctive antimicrobial treatment option. However, the possible side effects, like anaphylaxis, anemia, hearing loss and retinopathy have to be taken into account [211]. Inhibition of PHD and HIF stabilization holds potential as an adjunctive therapeutic agent. However, therapeutic stabilization of HIF1α has to be tailored individually. For instance, in a model of progressive pulmonary tuberculosis in BALB/c mice, the blockage of HIF1α worsened the disease during the early phase of infection, while it decreased bacterial load during late tuberculosis [212].

## Other bacteria thrive under hypoxia

Although hypoxia damages oxygen-dependent bacteria, induces HIF accumulation and activity, leads to increased release of pro-inflammatory cytokines and antimicrobial peptides, and prolongs the survival of neutrophils, it is not always detrimental to pathogens. First, several antimicrobial pathways such as NOS2 and PHOX require oxygen to produce their toxic compounds. Second, bacterial microorganisms frequently develop strategies to adapt to hypoxic conditions and to benefit from the low O_2_ level at the site of infection (Table 2).

In the case of *Shigella flexneri*, the local depletion of oxygen due to the aerobic respiration of the bacteria incapacitated the Ipa (invasion plasmid antigen)-dependent secretion of effector molecules and, thus, the virulence of *Shigella flexneri*, but at the same time promoted its local proliferation and the establishment of microcolonies within the gut tissue [34].

Furthermore, there is evidence that low oxygen conditions support the replication of *Salmonella enterica* serovar Typhimurium within macrophages. Hypoxia increased the activity of the *Salmonella* pathogenicity island 2 (SPI-2)-encoded type III secretion system and simultaneously impaired the activity of the antimicrobial enzymes NOS2 and PHOX ( [33]; Fig. 3; Table 2). At this stage, the role of HIF1α in macrophages during *Salmonella* pathogenesis is unexplored and requires further investigation. In addition, the mechanisms by which hypoxia increased SPI-2 activity are unclear and warrant further research. Moreover, the metabolic requirements that allow *Salmonella* survival and replication under low oxygen conditions remain elusive. The ability of *Salmonella* to generate all its metabolites from simple carbon, nitrogen, and sulfur sources [213] and the expression of high-affinity cytochromes [213] might be the prerequisites that enable *Salmonella* to thrive within low oxygen environments.

Low oxygen conditions were also reported to impair antimicrobial activity of macrophages directed against *E. coli* and *S. aureus* (Fig. 3) [214]. In addition, hypoxia impaired the regular function of mitochondria [214]. It is known that uncoupling of the electron transport chain and mitochondrial ROS production can contribute to the antimicrobial activity of macrophages [215–217]. Currently, it is, however, unclear which mitochondrial function is specifically impaired under hypoxic conditions.

## Concluding remarks

In this review, we attempted to shed light on the complex and diverse roles of oxygen in host–pathogen interaction. Low levels of oxygen trigger HIF-dependent pathways in host cells, which contribute to containment of bacterial replication and spreading. This is mainly mediated by upregulation of antimicrobial peptides or molecules and the alteration of the host immune response. In recent years, first examples demonstrated that bacterial containment under hypoxia can be accomplished by the depletion of metabolites caused by alteration of the host cell metabolism. A major challenge for future research will be to increase our understanding of the complex interplay between metabolites, immune responses and control of intruding pathogens.

Low oxygen environments can also be beneficial for the invading pathogen, as several antimicrobial effectors, such as PHOX and NOS2, depend on oxygen for production of their toxic products. Therefore, hypoxia might impede elimination of bacteria and might even result in increased replication under hypoxic condition or trigger bacterial dormancy. In this state, the bacteria can survive for a long period of time and are even protected from antibiotic treatment [218, 219]. As bacterial dormancy is the main cause for recurring and/or chronic infections, the hypoxia-induced bacterial containment might come with a high cost for the patient. It will be of major importance to increase our understanding of how bacterial dormancy and the re-entering into the replication mode are regulated under hypoxic conditions. As bacterial dormancy is a major cause for antibiotic inaccessibility, this knowledge might allow developing novel therapeutics.

The picture is even more complex, as the pathogens have evolved mechanisms to overcome and modulate the hypoxic conditions. Thus, the pathogens rely on oxygen sensors to adapt to hypoxic conditions. These sensors are mainly two component systems and allow the pathogen to transcriptionally react to changes in oxygen availability. Genes important for bacterial metabolism, for the induction of bacterial dormancy, and for virulence factors are transcribed. As different pathogens have distinct metabolic needs and virulence factors, the host–pathogen interaction under hypoxia has to be analyzed for each pathogen individually.

In addition, we are only beginning to understand the role of infection-induced HIF1α stabilization in host–pathogen interaction. This is due to the complex experimental set-up required to differentiate between the role of hypoxia/oxygen deficiency and the infection-induced HIF stabilization. Studies employing HIF stabilization agents may especially interesting to address this issue. From these studies, new targets and therapeutic strategies might emanate, which allow targeting of pathogens in their hypoxic niche. However, therapeutics targeting hypoxia and/or HIF also have to be tailored individually for each pathogen and site of infection and will not be available off the shelf.

## Abbreviations

ATP: Adenosine triphosphate
DCs: Dendritic cells
DSS: Dextran sulfate sodium
DMOG: Dimethyloxalylglycine
(Dos)R: Dormancy survival regulator
HIF: Hypoxia-inducible factors
IDO: Indoleamine 2,3-dioxygenase
iNOS or NOS2: Inducible or type 2 nitric oxide synthase
IFNγ: Interferon γ
IL: Interleukin
LDH-A: Lactate dehydrogenase-A
LPS: Lipopolysaccharide
MtrAB: *M. tuberculosis* Two-component regulatory system MtrA/MtrB
NET: Neutrophil extracellular traps
NO: Nitric oxide
OXPHOS: Oxidative phosphorylation
PHOX: Phagocyte NADPH-oxidase
PMN: Polymorphonuclear neutrophils
PHDs: Prolyl hydroxylases
PKM2: Pyruvate kinase M2
RNS: Reactive nitrogen species
ROS: Reactive oxygen species
SPI-2: *Salmonella* Pathogenicity island 2
TCA: Tricarboxylic acid cycle
VEGF: Vascular endothelial growth factor
